# Counteracting Effects of Cellular Notch and Epstein-Barr Virus EBNA2: Implications for Stromal Effects on Virus-Host Interactions

**DOI:** 10.1128/JVI.01431-14

**Published:** 2014-10

**Authors:** Martin Rowe, Sweta Raithatha, Claire Shannon-Lowe

**Affiliations:** School of Cancer Sciences, The University of Birmingham, Birmingham, United Kingdom

## Abstract

A number of diverse environmental cues have been linked to B lymphocyte differentiation and activation. One such cue, Notch-2, may be particularly relevant to the biology of infection with Epstein-Barr virus (EBV), which colonizes the B cell compartment. Activated Notch and EBV nuclear antigen 2 (EBNA2) both function as transcriptional activators by virtue of their interactions with the transcription factor RBP-Jκ. Although EBNA2 and activated Notch appear to have partially overlapping functions, we now report that activated Notch counteracts a crucial EBNA2 function both in newly infected primary B cells and in lymphoblastoid cell lines (LCLs). EBNA2 is directly responsible for the initiation of transcription of the majority of EBV proteins associated with type III latency, leading to the outgrowth of LCLs. One of the key proteins driving this outgrowth is latent membrane protein 1 (LMP1), which is regulated by an EBNA2-responsive element within its ED-L1 promoter. Activation of Notch-2 via Delta-like ligand 1 inhibits EBNA2-mediated initiation of LMP1 transcription. Furthermore, ligated Notch-2 also efficiently turns off LMP1 expression from the ED-L1 promoter in LCLs already expressing LMP1. Modulation of EBV gene expression by Notch was not confined to EBNA2-dependent events. Activated Notch-2 also inhibited EBV entry into the lytic cycle in a B cell non-Hodgkin's lymphoma line by upregulating the cellular transcription factor Zeb2, which represses the transcription of BZLF1. These results support the concept that *in vivo*, cumulative signals from the microenvironment downregulate EBV gene expression in B cells to the latency 0 gene expression profile observed in B cells entering the peripheral blood.

**IMPORTANCE** Experimental infection of resting B cells by Epstein-Barr virus leads to the growth transformation program of virus gene expression and the outgrowth of lymphoblastoid cell lines. Previous studies at the single-cell level revealed complex cellular and viral signaling networks regulating transcription of the viral genome. This study demonstrates that viral gene expression can also be radically altered by molecules expressed on stromal cells in the microenvironment of lymphoid tissue, specifically, Delta-like ligand 1 on stromal cells ligating Notch-2 on infected B cells. Activation of Notch interferes with the transactivation function of EBNA2, downregulates the expression of LMP1 and LMP2a, and inhibits the activation of lytic virus replication in a B cell non-Hodgkin's lymphoma line by preventing expression of BZLF1. The significance of these observations is that they indicate new mechanisms whereby the microenvironment in normal lymphoid tissue may facilitate the repression of viral gene expression, enabling establishment of true latency in memory B cells.

## INTRODUCTION

The Notch pathway is an evolutionarily conserved intercellular signaling pathway that transduces signals between adjacent cells and plays an essential role in cell fate determination during development, tissue homeostasis, and stem cell maintenance (1). The intercellular signaling is mediated by four conserved receptors (Notch 1 to 4) and two conserved families of Notch ligands (Delta-like and Jagged), located on the plasma membrane. Activation of Notch receptors on the cell surface requires direct contact between the extracellular domain of a Notch receptor with one of the Notch ligands expressed on an adjacent cell. Notch ligation triggers a regulated sequence of proteolytic cleavages (2, 3) which releases the intracellular Notch (ICN) fragment to translocate to the nucleus, interact with the DNA binding protein RBP-Jκ, and function as a transcriptional activator (4–6).

Notch signaling events have been extensively studied during T lymphopoiesis, where they play a crucial role during both early development and differentiation into discrete effector cell compartments (7, 8). Notch is also implicated in regulation of multiple stages of B cell development. It is now evident that Notch signaling is crucial to generate the marginal zone B cell compartment located within the spleen (9–11), and there is compelling evidence that Notch may help shape the antibody repertoire (12). The gene for Notch-2 is the primary Notch gene expressed by peripheral B cells and predominantly interacts with Delta-like ligand 1 (DLL1) (9–11).

Epstein-Barr virus (EBV), which is carried by the vast majority of all adults worldwide, establishes a lifelong persistent infection through colonizing the B lymphocyte compartment as a latent infection. It has been postulated (13) that EBV exploits the normal physiology of B cell differentiation to regulate its own gene expression. In addition to sharing a general feature of all herpesviruses, which is the ability to expand the virus-infected cell pool by periodic lytic replication to generate new infectious virions, EBV additionally has the potential to expand the pool of virus-infected cells through growth transformation of B lymphocytes. *In vitro*, this property is manifest following infection of purified resting B cells, which results in the establishment of continuously proliferating lymphoblastoid cell lines (LCLs). B cell infection follows a highly regulated cascade of viral gene expression, starting with expression of the Epstein-Barr virus nuclear antigens (EBNAs) EBNA2 and EBNA-LP (14). EBNA2 plays a pivotal role in B cell infection through activating the transcription of almost all the viral genes that are expressed during the growth transformation of B cells, notably, the viral latent membrane proteins (LMPs) LMP1, LMP2A, and LMP2B (15, 16), and the C promoter (Cp)-driven expression of the nuclear antigens EBNA1, EBNA3A, EBNA3B, and EBNA3C (17, 18). EBNA2 cannot bind to the viral DNA itself to activate gene expression but interacts with RBP-Jκ (19–21), which tethers EBNA2 to EBNA2 response elements within promoter regions of DNA. EBNA2 also activates the transcription of cellular genes, including CD23, MYC, and CCR7 (21, 22).

Given that both ICN (intracellular or activated Notch) and EBNA2 function as transcriptional activators by virtue of their interaction with RBP-Jκ, EBNA2 could be regarded as a functional homologue of an active Notch receptor, albeit EBNA2 is constitutively active and functions independently of a cell surface ligand (23–25). In EBNA2-negative Burkitt lymphoma (BL) cell lines, mouse ICN-1 is able to upregulate some EBNA2 viral targets, including LMP2A, although not others, such as LMP1. Similarly, ICN-1 is able to upregulate the EBNA2 cellular target CD21 but not CD23. Additionally, both ICN-1 and EBNA2 downregulate immunoglobulin M (IgM) expression, indicating that Notch has both positive and negative regulatory functions. However, analysis of the effect of ICN-1, ICN-2, and EBNA2 expression on genome-wide transcription in EBV-infected B cell lines revealed profound differences in the regulation of target genes, with only a small cluster of genes being concordantly regulated (26). EBNA2 appears to be more efficient in upregulating genes involved in proliferation, survival, and chemotaxis, whereas ICN-1 and ICN-2 are more potent at inducing genes associated with development and differentiation.

Although it is now known that EBNA2 and Notch regulate largely nonoverlapping gene sets, it is not yet clear to what extent Notch might modulate EBV infection. This is a fundamental question that is relevant for understanding virus persistence *in vivo*, where virus-infected cells are exposed to intercellular signaling molecules within stromal cell microenvironments.

In the present report, we examine how Notch ligation, via interaction with stromal cells producing the Notch ligand Delta-like ligand 1, regulates EBV gene expression in both latent and lytic infection of B cells. We show that Notch signaling modulates latent viral gene expression, most notably by inhibiting EBNA2-dependent expression of LMP1, and can block entry into the lytic cycle via an EBNA2-independent Zeb2-mediated block of BZLF1 expression.

## MATERIALS AND METHODS

### Ethics.

Ethical approval for the use of primary B cells and bone marrow was granted by the Solihull Local Research Ethics Committee (06/Q2706/71) and the South Birmingham Research Ethics Committee (07/H128/62).

### Cells and cell lines.

Primary B cells were isolated from peripheral blood using CD19 Dynabeads and CD19 Detachabeads (Invitrogen), as previously described (27).

Cultures of mouse thymic stromal cells (OP9) expressing the human Delta-like ligand 1 (OP9-DLL1 cells) or control green fluorescent protein (GFP; OP9-GFP cells) (kindly provided by Graham Anderson, The University of Birmingham) were maintained for up to 20 passages in Opti-MEM medium (Gibco, Life Technologies) with 20% fetal calf serum (FCS).

BNHL-1 cells were derived from a bone marrow sample from a patient being treated for low-grade B cell non-Hodgkin's lymphoma (NHL). Cells of the BNHL-1 cell line were phenotypically the same as cells of the low-grade B cell NHL observed in the patient. These cells were infected with EBV and were continually maintained on the OP9-DLL1 cells, unless specified otherwise. An EBV-negative counterpart was maintained in the same manner.

293 cells containing recombinant wild-type EBV strain 2089 were maintained in RPMI, 10% FCS, and 100 μg/ml hygromycin. Akata cells containing recombinant wild-type EBV Akata were maintained in RPMI, 10% FCS, and 50 ng/ml G418.

### Virus preparations and infection experiments.

Preparations of recombinant wild-type EBV Akata with a GFP insert (28) were generated by inducing the virus producer B cell line with 0.1% goat anti-human polyclonal IgG (MP Biomedicals). Preparations of recombinant wild-type EBV 2089 with a GFP insert (29) were made from 293 cells carrying the recombinant EBV B95.8 genome, as previously described (27). Encapsidated and enveloped virus was purified from the culture supernatants by centrifugation on an OptiPrep (Axis Shield) self-generated gradient (30) and quantitated by quantitative PCR (qPCR) of a single-copy gene, *BALF5*, as previously described (27). The multiplicity of infection (MOI) of the virus used in infection experiments was defined as the number of EBV genomes in the purified virus preparations divided by the number of target cells in culture.

EBV infection of primary B cells isolated from peripheral blood was carried out at an MOI of 50, as described previously (27).

### qRT-PCR for EBV and cellular transcripts.

Total RNA was isolated from cells using a NucleoSpin II RNA purification kit (Macherey-Nagel) according to the manufacturer's instructions. One microgram of RNA was further digested with DNase (DNA-free; Ambion) according to the manufacturer's instructions. For quantitative real-time PCR (qRT-PCR) of EBV and cellular transcripts, 400 ng of RNA was reverse transcribed in a 20-μl reaction volume using qScript cDNA SuperMix (Quanta-Biosciences) according to the manufacturer's instructions.

Quantitative RT-PCRs for EBV transcripts were performed using EBV gene-specific primers as previously reported (31). Briefly, 20 ng of cDNA was added to each qRT-PCR mixture to quantify W promoter (Wp)- and C promoter (Cp)-initiated EBNA mRNAs; Y-U-K-spliced EBNA1 mRNA; Q promoter (Qp)-initiated Q-U-K-spliced EBNA1 mRNA; and LMP1, LMP2a, BZLF1, and gp350 mRNAs.

Absolute quantitation of LMP1 and LMP2a transcripts was performed using primers for total LMP1 (31) and for LMP1 originating from the promoter (LT-R1) located in the terminal repeats (TRs): primer LMP1 (5′-CGTAGCCGCCCTACATAAGC; in the terminal repeats), primer LMP1 (3′-CCCCTCTCAAGGTCGTGTTC; in exon 1), and the LMP1 probe (CCTCAGGGCAGTGTGTCAGGAGCA; in exon 1). A plasmid construct containing the relevant amplicons for total LMP1, LMP1 derived from the LT-R1 promoter, and beta-2-microglubulin (B2M) of known copy number was diluted to give a standard curve from 10^5^ copies down to 1 copy in 10-fold dilutions. The standards and samples were amplified (as reported previously 31), and the number of LMP1 transcripts per cell was determined.

Relative quantitative RT-PCRs for cellular transcripts were performed using TaqMan assays for each mRNA transcript (Life Technologies). Briefly, 20 ng of cDNA was added to each qRT-PCR mixture to quantify Oct-1, Oct-2, Zeb1, Zeb2, MYC, and PU.1. Each qRT-PCR assay was performed by determination of the level of expression relative to that in a suitable reference cell line, for which the result was given an arbitrary value of 1, and then the level of expression was normalized against the level of expression of the B2M housekeeping gene. Oct-1 expression was determined relative to that in Jurkat cells, Oct-2 expression was determined relative to that in Ramos cells, Zeb1 expression was determined relative to that in Jurkat cells, and Zeb2 expression was determined relative to that in HeLa cells, as suggested by the antibody manufacturer.

### Detection of EBV and cellular proteins by immunofluorescence.

EBV-infected cells were fixed in 4% paraformaldehyde for 20 min at room temperature and centrifuged onto poly-l-lysine-coated slides (Sigma) using a Shannon cytospin apparatus. The cells were permeabilized in 0.5% Triton X-100 and stained for the EBV proteins EBNA1 (monoclonal antibody R4; a kind gift from Laurie Frappier), BZLF1 (monoclonal antibody BZ.1 [32]), EBNA2 (monoclonal antibody PE2 [33]), LMP1 (monoclonal antibody CS.1-4 [34]), gp350 (monoclonal antibody 72A1 [35]), and viral capsid antigen (VCA; Chemicon). All nonconjugated primary antibodies were detected with the anti-mouse or anti-rabbit secondary antibodies, as appropriate, labeled with Alexa Fluor 488 or 594 (Life Technologies).

### Western blot analysis.

Cells were lysed in urea buffer (9 M urea, 50 mM Tris, pH 7.5), sonicated, and quantitated against bovine serum albumin standards using a Bio-Rad DC protein assay. The cell lysates were diluted in sample buffer to give a final concentration of 1 mg/ml protein in 2% sodium dodecyl sulfate (SDS), 62.5 mM Tris-HCl, pH 6.8, 10% glycerol, 0.2 M sodium 2-mercaptoethanesulfonate, 0.002% bromophenol blue and then heated to 100°C for 5 min. Solubilized proteins were separated by SDS-electrophoresis on 4 to 12% gradient bis-Tris NuPAGE minigels with MOPS (morpholinepropanesulfonic acid) running buffer (Life Technologies). Following electroblotting onto polyvinylidene difluoride membranes (Life Technologies) and blocking in 5% fat-free milk and 1% Tween 20 in phosphate-buffered saline, pH 7.2, the membranes were incubated with primary antibodies overnight at 4°C. The antibodies to Oct-1 (antibody C-21), Oct-2 (antibody C-20), Zeb1 (antibody H-102), and Zeb2 (antibody H-260) were all used at 1 μg/ml, and calregulin antibody was used at 0.2 μg/ml (Santa Cruz Biotechnology). The antibodies to Notch-1 (antibody D3B8), Notch-2 (antibody D76A6), Notch-3 (antibody 8G5), and Notch-4 (antibody L5C5) (Cell Signaling Technology) were all used at a dilution of 1/1,000. The HLA class I antibody (HC10) was used at a dilution of 1/1,000 (36), and the histone H3 antibody (pS10; Life Technologies) was used at a dilution of 1/1,000. Positive controls consisted of Notch-1 in Jurkat cells, Notch-2 in 293 cells, Notch-3 in K562 cells, and Notch-4 in Jurkat cells.

### Isolation of nuclei.

Nuclei were isolated using a Nuclei EZ Prep nucleus isolation kit (Sigma) according to the manufacturer's protocol. The numbers of whole cells and nuclei were recorded to ensure that exactly the same numbers of cells used for the loading control were prepared for Western blotting.

### Inhibition and knockdown experiments.

To inhibit Notch signaling, EBV-infected B cells were incubated in culture medium with 5 μM γ-secretase inhibitor IX (Calbiochem) for from 24 h to 6 days. The medium was replaced every 2 days with fresh inhibitor. Matched treated and nontreated cells were harvested every 24 h, RNA was extracted, and protein samples were prepared for analysis of the downstream effects of inhibitor treatment.

Knockdown of Zeb2 expression in BNHL-1 cells was achieved using short hairpin (shRNA) lentiviral vectors. BNHL-1 cells were transduced with lentivirus Zeb2 shRNA and lentivirus control shRNA (Santa Cruz Biotechnology) according to the manufacturer's instructions. Briefly, 0.25 × 10^6^ cells were plated in complete medium containing 5 μg/ml Polybrene and 250 μl lentivirus and incubated overnight. To determine the transduction efficiency, control lentivirus expressing GFP (Santa Cruz) was used exactly as described above, and the cells were analyzed by flow cytometry after 24 h incubation. Cells were harvested every 2 days postransduction and assayed for Zeb2 knockdown by Western blotting.

## RESULTS

The initial set of experiments was performed on the BNHL-1 cell line, cells of which were established and maintained on mouse thymic stromal cells expressing the human Notch ligand Delta-like ligand 1 (OP9-DLL1 cells). Serendipitously, when these cells were experimentally infected with EBV, they revealed remarkable Notch-dependent regulation of viral gene expression.

### EBV-infected BNHL-1 cells adopt a strict latency I phenotype when grown on OP9 stromal cells expressing human Delta-like ligand 1.

**FIG 1:**
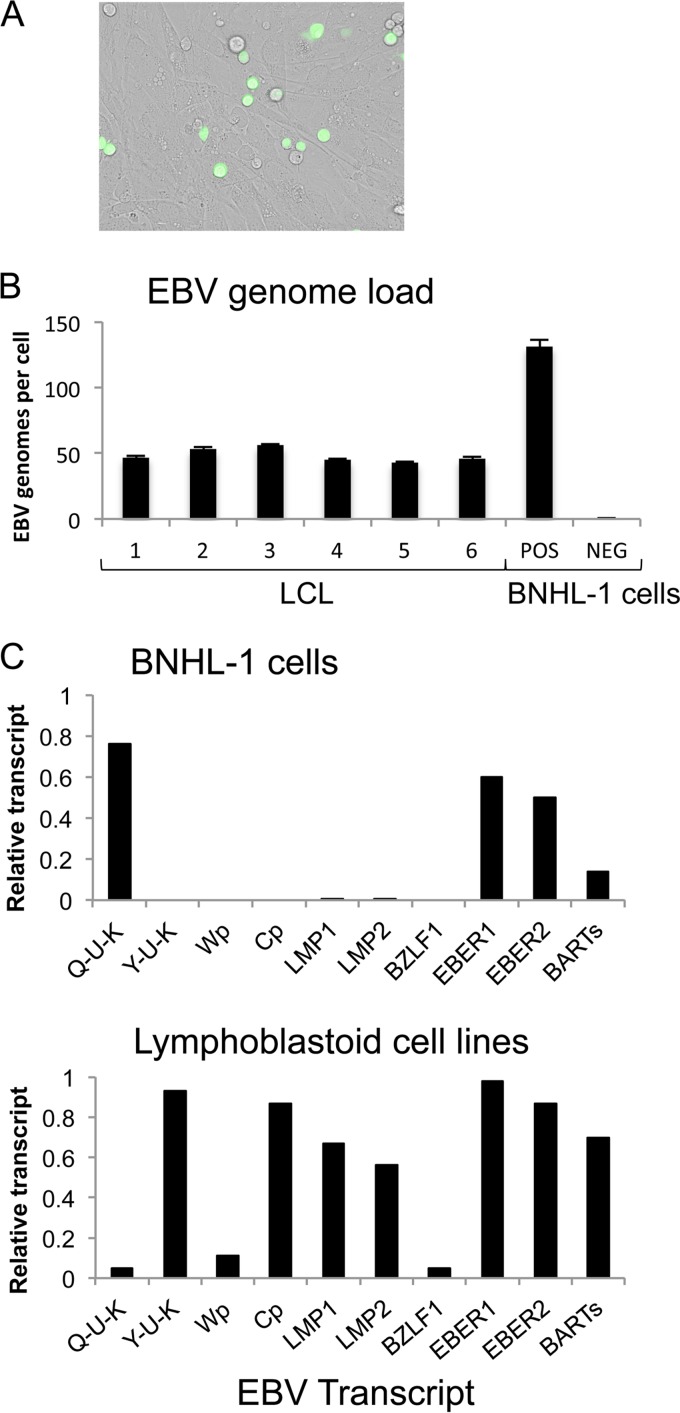
Generation of a BNHL-1 cell line with a strict latency I phenotype. (A) Photomicrograph of EBV-infected (GFP-positive) and uninfected (GFP-negative) BNHL-1 cells cocultured on mouse thymic stromal cells expressing the human Delta-like ligand 1 (OP9-DLL1 cells). (B) EBV load of GFP-positive (POS) and GFP-negative (NEG) BNHL-1 cells compared to EBV load of six individual LCLs. The results of assays performed in triplicate are shown. (C) Quantitation of relative levels of EBV mRNA transcripts by qRT-PCR in LCLs (top) and BNHL-1 cells (bottom) cocultured with OP9-DLL1 cells. The histograms show for each EBV transcript the amount of mRNA transcripts relative to that for a suitable reference cell line, which was given an arbitrary value of 1.

At day 8 following explantation of patient bone marrow-derived cells onto OP9-DLL1 stroma, the BNHL-1 cells were infected with recombinant EBV Akata. More than 50% of the lymphoma cells were infected, as judged by the expression of GFP (Fig. 1A). Following cell sorting into GFP-positive and GFP-negative B cell populations, measurement of the EBV genome load by qPCR confirmed EBV infection with approximately 130 genomes per cell in only the GFP-positive cells (Fig. 1B).

Examination of EBV gene transcription in the BNHL-1 cells revealed a pattern of expression that was consistent with a restricted latency I rather than the latency III that is characteristically observed in the LCLs established following infection of normal primary B cells. Thus, we observed EBNA1 transcription, plus that of the EBER and BART genes, only from the Q promoter (Qp) across the Q-U-K splice; expression of other latent genes was suppressed, as was expression of the BZLF1 immediate early lytic cycle gene (Fig. 1C, top). This form of EBV gene expression was quite distinct from latency III, or the growth transformation program, that is observed in normal LCLs (Fig. 1C, bottom). In LCLs, EBNA transcription is initially driven from the W promoter (Wp) and then switches to the C promoter (Cp), allowing expression of all 6 EBNA genes. In this Wp/Cp form of latency, known as latency III, the EBNA1 transcripts are generated via the Y-U-K splice, as opposed to the Q-U-K splice observed in latency I. In addition to the complete repertoire of EBNAs, latency III is associated with EBNA2-induced expression of the LMP1 and LMP2A transcripts that were repressed in the EBV-infected BNHL-1 cells.

### Removal from OP9-DLL1 stromal cells triggers the EBV lytic cycle in BNHL-1 cells.

**FIG 2:**
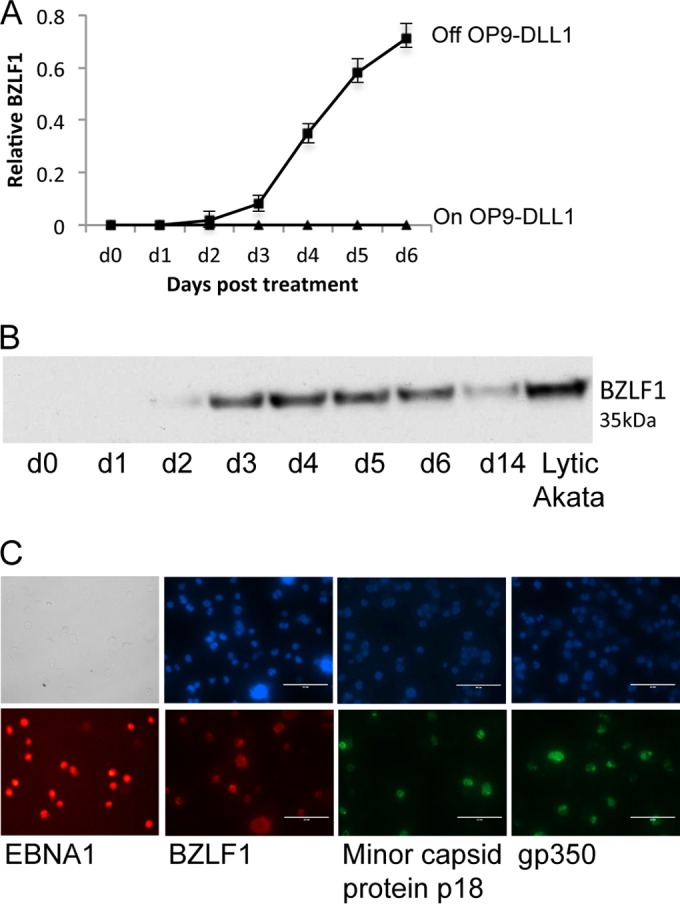
Removal of EBV-infected BNHL-1 cells from the OP9-DLL1 stromal cells triggers the EBV lytic cycle. (A) Quantitation of relative levels of EBV BZLF1 mRNA transcripts by qRT-PCR in EBV-infected BNHL-1 cells cultured on or off OP9-DLL1 stromal cells. An example graph of the level of expression of BZLF1 transcripts normalized to that in lytic Akata BL cells as the reference cell line is shown. Each assay was performed in triplicate. d, day. (B) Western blot analysis of BZLF1 expression in BNHL-1 cells following removal from OP9-DLL1 stromal cells probed with a BZLF1-specific monoclonal antibody (BZ1). (C) Single-cell immunofluorescence of BNHL-1 cells 6 days after removal from OP9-DLL1 stromal cells. The cells were probed with antibodies for both immediate early (BZLF1) and late (minor capsid protein gp350) lytic cycle proteins, revealing that 20 to 40% of cells removed from OP9-DLL1 stromal cells entered the lytic cycle.

The restricted pattern of latent EBV gene expression in newly infected BNHL-1 B cells was unexpected, since EBV-negative Burkitt lymphoma (BL) lines typically initially establish a latency III pattern of gene expression when experimentally infected with EBV, in contrast to the latency I pattern characteristically displayed by tumor cell lines derived from EBV-positive BL biopsy specimens. However, following extended culture, latency I can be established in a subset of BL clones (37). We hypothesized that coculture of BNHL-1 cells with stromal cells might be modulating viral gene expression. To test this possibility, the infected BNHL-1 cells were removed from the OP9-DLL1 cells and aliquots were harvested daily thereafter to determine if the pattern of EBV gene expression changed. In fact, the pattern of latent gene expression was essentially unchanged following removal of BNHL-1 cells from the OP9-DLL1 stroma (data not shown). Remarkably, however, removal of DLL1/Notch signaling led to a striking disruption of latency with induction of BZLF1 and the lytic cycle. Figure 2A shows the results of qRT-PCR assays of BZLF1 mRNA transcription over 6 days following removal from OP9-DLL1 cells. The level of BZLF1 transcription in EBV-positive BNHL-1 cells was compared to the level of BZLF1 mRNA observed in Akata cells at 12 h following induction of the lytic cycle by ligation of its B cell receptor. BZLF1 mRNA levels increased rapidly from 3 days onwards to 70% of the levels observed in Akata BL cells induced into the lytic cycle. This induction of the lytic cycle in infected BNHL-1 cells following removal of stromal interactions was confirmed by immunoblotting for BZLF1 protein (Fig. 2B) and by single-cell immunofluorescence (Fig. 2C). In replicate experiments, the proportion of infected BNHL-1 cells expressing BZLF1 by day 6 ranged from 20% to 40%. Interestingly, the induction of the lytic cycle in the BNHL-1 cells removed from OP9-DLL1 stromal cells progressed efficiently to virus production, as shown by the immunofluorescence for capsid protein and gp350 (Fig. 2C), with at least half of the cells that initiated lytic replication expressing these late viral structural genes.

BZLF1 expression following removal from the OP9-DLL1 cells was presumed to result from the removal of DLL1/Notch signaling. To discount the possibility that other cytokines released from the OP9-DLL1 cells might be modulating EBV gene expression, we first removed the infected BNHL-1 cells from the OP9-DLL1 cells and cultured them either in conditioned medium from the OP9-DLL1 cells or in fresh medium and then compared the levels of BZLF1 mRNA transcription. We found no difference in either the rate of cell growth or the level of BZLF1 expression between cells in fresh and conditioned medium (data not shown). We then examined whether BZLF1 transcription was regulated by contact with stromal cells or by the Delta-like ligand 1. We therefore compared EBV gene transcription in BNHL-1 cells cocultured with the OP9-DLL1 cells or with control OP9-GFP stromal cells or cultured off stroma. We found that BZLF1 mRNA transcription was inhibited only when the cells were cocultured on OP9-DLL1 cells; when the cells were cocultured with OP9-GFP cells, BZLF1 transcription was initiated at the same rate seen when the cells where removed from the stroma (data not shown).

### BZLF1 expression is regulated by Notch-2 ligation.

**FIG 3:**
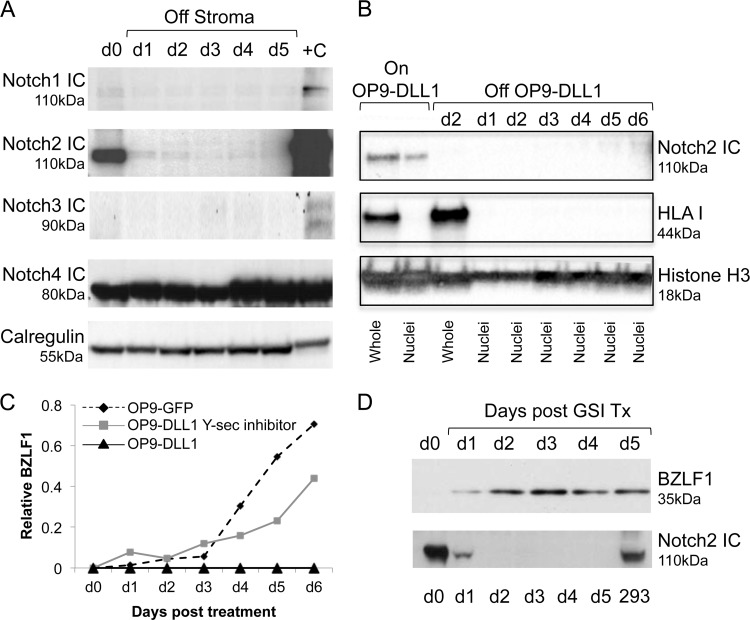
Notch-2 regulates entry into the lytic cycle. (A) Western blot analysis of BNHL-1 cells cocultured with OP9-DLL1 stromal cells (day 0) or cultured for up to 5 days following removal from the OP9-DLL1 cells. Each blot was probed with antibodies to truncated intracellular (IC) Notch-1, -2, -3, or -4. Intracellular Notch-2 was observed when it was ligated by DLL1, but its levels rapidly dropped by 24 h following its removal. Positive controls consisted of Notch-1 in Jurkat cells, Notch-2 in 293 cells, Notch-3 in K562 cells, and Notch-4 in Jurkat cells. +C, positive control. (B) Western blot analysis of whole-cell lysates and nuclei of BNHL-1 cells cultured on and off OP9-DLL1 cells. The blot was probed for ICN-2 for detection of activated Notch-2, HLA class I for detection of plasma membrane components, and for histone H3 for detection of components of the nuclei only. This shows that activated Notch is present only in the nucleus of cells during cocultivation of BNHL-1 cells on OP9-DLL1 cells. (C) Quantitation of relative levels of BZLF1 mRNA transcripts in BNHL-1 cells cocultured with OP9-DLL1 stromal cells, OP9-GFP stromal cells, and OP9-DLL1 stromal cells with a γ-secretase (γ-Sec) inhibitor to inhibit the cleavage of Notch into the activated intracellular Notch. Inhibition of Notch activation by the γ-secretase inhibitor resulted in the induction BZLF1. Each assay was performed in triplicate. (D) Western blot analysis of BNHL-1 cells cocultured on OP9-DLL1 cells and treated with the γ-secretase inhibitor. The blots were probed with antibodies to BZLF1 and Notch-2. BZLF1 was expressed following treatment (Tx) with the γ-secretase inhibitor (GSI) (top), and inhibitor treatment resulted in undetectable intracellular Notch-2 (bottom).

The aforementioned experiments strongly implicated Delta-like ligand 1 as an inhibitor of BZLF1 expression. Given that Delta-like ligand 1 is a pan-Notch ligand, we then asked which Notch on the BNHL-1 cell surface was interacting with DLL1 mediating the signal transduction. Western blotting analyses for intracellular Notch-1 (ICN-1), ICN-2, ICN-3, and ICN-4 were performed on the infected BNHL-1 cells during OP9-DLL1 cell coculture and following removal from OP9-DLL1 cells (Fig. 3A). These experiments showed that ICN-1 and ICN-3 were poorly or not detected in BNHL-1 cells, while ICN-4 was detected at high levels throughout the time course, suggesting constitutive activation. However, ICN-2 was detected only when cocultured with OP9-DLL1 cells (day 0). Within 24 h of removal from the OP9-DLL1 cells, the level of ICN-2 had decreased to almost undetectable levels. These data show that BNHL-1 Notch-2 is ligated by DLL1 on the stromal cell surface and that removal of the BNHL-1 cells from the stromal cells abolished Notch-2 signaling.

To formally confirm that Notch was activated, the BNHL-1 cells were cocultured with OP9-DLL1 cells or removed from OP9-DLL1 cells for 6 days. The nuclei were isolated from the cocultured cells and every day following removal from OP9-DLL1 cells. Western blot analysis was performed on whole-cell lysates and nuclear lysates, with equal numbers of cells being added to each lane. The blots were probed for Notch to ensure that it was detectable only when cells were cocultured; for HLA class I, which is not found in the nucleus; and for histone H3, which is found only in the nucleus. The results confirmed that activated (nuclear) Notch2 is detected only in the nucleus when the BNHL-1 cell line is cocultured with OP9-DLL1 cells.

To confirm that BZLF1 was being regulated by Notch-2 ligation, the BNHL-1 cells on OP9-DLL1 cells were treated with an inhibitor of γ-secretase, the enzyme which cleaves Notch on the plasma membrane and releases ICN to translocate to the nucleus. The BNHL-1 cells were cocultured with OP9-DLL1 cells and treated with the γ-secretase inhibitor on day 0 and then subsequently every 2 days for 6 days. Cell aliquots were harvested daily for extraction of RNA and protein. Figures 3B and C show the results of qRT-PCR and Western blot analysis. In the γ-secretase inhibitor-treated cells, BZLF1 transcription was observed from day 1 posttreatment and increased over the 6 days (Fig. 3C). Similarly, the BZLF1 protein was observed from day 1 posttreatment by Western blotting (Fig. 3D, top). Importantly, a Western blot assay for ICN-2 performed on the same cells confirmed that the cleavage of Notch-2 was inhibited by treatment with the inhibitor of γ-secretase (Fig. 3D, bottom). These data confirm that BZLF1 expression is regulated by Notch-2 ligation.

### Zeb2 is regulated by Notch-2 ligation.

**FIG 4:**
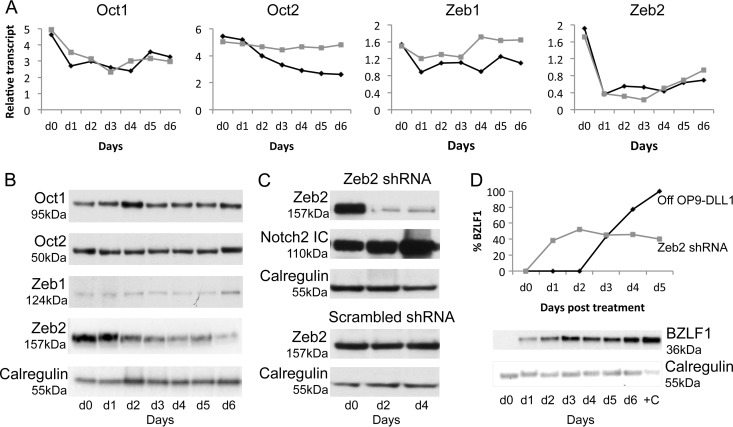
Zeb2 is regulated by Notch ligation. (A and B) Analysis of Oct-1, Oct-2, Zeb1, and Zeb2 expression in BNHL-1 cells by quantitation of relative levels and Western blotting. (A) BNHL-1 cells were either removed from OP9-DLL1 stromal cells (black line) or cocultured on OP9-DLL1 stromal cells with a γ-secretase inhibitor (gray line). Only the levels of the Zeb2 mRNA transcripts were reduced following removal from stromal cells and following γ-secretase inhibitor treatment. Each assay was performed in triplicate. (B) The findings described for panel A are also reflected in protein levels, where only the Zeb2 protein levels were reduced following removal from stromal cells (or γ-secretase inhibitor treatment; not shown). (C) Zeb2 knockdown by shRNA. BNHL-1 cells were transduced by lentiviruses expressing shRNA to knock down the levels of Zeb2 expression or scrambled shRNA and maintained on OP9-DLL1 stromal cells. (Top two panels) Zeb2 knockdown in the presence of Notch ligation; (fourth panel) Zeb2 following transduction with scrambled shRNA. (D) Quantitation of relative levels of BZLF1 mRNA and protein in BNHL-1 cells maintained on OP9-DLL1 stromal cells following Zeb2 knockdown. BZLF1 is expressed in the absence of Zeb2.

To determine how Notch inhibits BZLF1, we examined whether known regulators of BZLF1 or BRLF1 were themselves regulated by Notch. In the first instance, we examined Oct-1 and Oct-2. Oct-1 binds to the BRLF-1 promoter to induce BRLF1 transcription and the subsequent lytic cycle (38). Conversely, Oct-2 binds to the BZLF1 protein and inhibits the lytic cycle (39). To determine if Oct-1 and Oct-2 are regulated by Notch ligation, we examined their mRNA (Fig. 4A) and protein (Fig. 4B) expression levels in BNHL-1 cells over a 6-day period following removal from OP9-DLL1 cells and in γ-secretase inhibitor-treated BNHL-1 cells on OP9-DLL1 cells. No difference in the transcription of Oct-1 mRNA in BNHL-1 cells off OP9-DLL1 cells or following inhibition by γ-secretase was observed (Fig. 4A, first graph). Furthermore, the concentration of Oct-1 protein remained constant over the entire time course (Fig. 4B, top). While the level of transcription of Oct-2 mRNA in BNHL-1 cells decreased by up to 2-fold following removal from OP9-DLL1 cells, this reduction was not reflected in BNHL-1 cells remaining on OP9-DLL1 cells but treated with the γ-secretase inhibitor, which suggests that the effect of stroma on Oct-2 mRNA expression in BNHL-1 cells is not specific to the DLL1-Notch interaction (Fig. 4A, second graph). Furthermore, the levels of the Oct-2 protein were unchanged (Fig. 4B, second panel). Together, these data suggest that neither Oct-1 nor Oct-2 is directly regulated by Notch ligation.

Next, we examined Zeb1 and Zeb2, both of which are reported to interact with the BZLF1 promoter and inhibit the lytic cycle in model systems (40). As described above, we examined the expression of Zeb1 and Zeb2 mRNA and protein expression levels in BNHL-1 cells off OP9-DLL1 cells or in BNHL-1 cells on OP9-DLL1 cells but treated with the γ-secretase inhibitor. Little difference in the transcription of Zeb1 mRNA in BNHL-1 cells off OP9-DLL1 cells or following inhibition of γ-secretase was apparent (Fig. 4A, third panel), and the concentration of Zeb1 protein remained constant (Fig. 4B, third graph). In marked contrast, the transcription of Zeb2 mRNA was reduced by up to 6-fold following removal from OP9-DLL1 cells and similarly following inhibition of γ-secretase while on OP9-DLL1 stroma (Fig. 4A, fourth graph). Furthermore, the effect on Zeb2 mRNA was paralleled by a reduction in the levels of Zeb2 protein detected by Western blotting (Fig. 4B, bottom). This strongly suggests that Zeb2 is regulated by Notch ligation.

To confirm that Zeb2 was regulating the expression of BZLF1 in this system, we performed Zeb2 knockdown experiments in BNHL-1 cells during coculture with OP9-DLL1 cells. Using a lentivirus shRNA construct containing 3 target-specific constructs or a control lentivirus containing a scrambled shRNA sequence, we transduced the BNHL-1 cells with each of the lentiviruses while the BNHL-1 cells remained on OP9-DLL1 cells to ensure that Notch ligation was still intact during the knockdown. Figure 4C shows Western blots for Zeb2 of BNHL-1 cells transduced with the Zeb2 shRNA or the control scrambled shRNA. The level of Zeb2 protein on days 2 and 4 postinfection decreased by up to 70% compared to that for cells transduced with the scrambled shRNA control. On the same cells, the unaltered presence of ICN-2 was confirmed (Fig. 4C). Having confirmed the successful knockdown of Zeb2, we asked if Zeb2 was responsible for the inhibition of BZLF1 transcription. As shown in Fig. 4D, in the presence of ICN-2, the knockdown of Zeb2 induced the transcription of BZLF1. Together, the data in Fig. 4 indicate that Notch ligation upregulates Zeb2, which itself directly represses BZLF1.

### Notch ligation inhibits virus BZLF1 expression in primary B cell infection.

**FIG 5:**
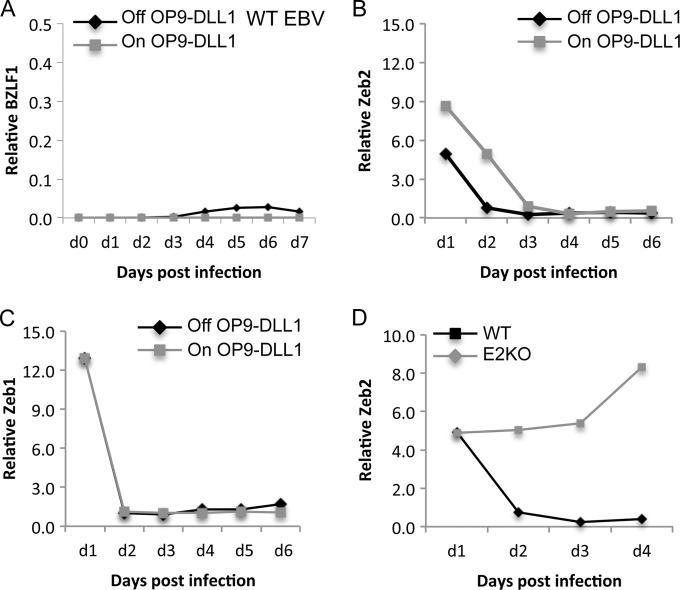
Notch ligation inhibits BZLF1 in primary B cell infection. (A) Quantitation of relative levels of BZLF1 mRNA expression in primary B cell infection. Primary resting B cells were infected and cocultured on and off OP9-DLL1 stromal cells. The cells cultured off stroma or on OP9-GFP stromal cells expressed a very small amount of BZLF1 transcripts, which were inhibited when the B cells were cultured on OP9-DLL1 stromal cells. WT, wild type. (B and C) Quantitation of relative levels of Zeb1 and Zeb2 mRNA transcripts in primary B cell infection on and off OP9-DLL1 stroma. The levels of the Zeb1 and Zeb2 mRNA transcripts dropped rapidly both in the presence and in the absence of the Notch ligand, albeit the rate of drop of Zeb2 was lower in the presence of the Notch ligand. (D) Quantitation of relative levels of Zeb2 mRNA transcripts in primary B cell infection with recombinant wild-type EBV and EBV with an EBNA2 deletion (EBNA2 knockout [E2KO]) off OP9-DLL1 stromal cells. The Zeb2 mRNA transcript levels did not drop in cells infected with the virus with the EBNA2 deletion compared to the levels in cells infected with wild-type virus. The same was observed when the cells were cultured on OP9-DLL1 cells. Each assay was performed in triplicate on three biological replicates. Representative plots are shown.

We next wanted to examine if Notch ligation would inhibit the expression of BZLF1 in a primary B cell infection. We infected newly isolated resting B cells with wild-type virus and immediately cocultured the B cells on the OP9-DLL1 cells or the control OP9-GFP cells. Figure 5A represents the results of qRT-PCR of BZLF1 mRNA transcription for four independent B cell infection experiments over 7 days postinfection. A very low level of BZLF1 mRNA transcription was detected in cells cocultured on the OP9-GFP cells, which is typical of what we normally observe in B cell infection experiments, but was not detected in cells cocultured on the OP9-DLL1 cells. In extended experiments of up to 30 days, BZLF1 inhibition was maintained (data not shown). However, if an established LCL with a small but defined level of BZLF1 expression was cocultured with the OP9-DLL1 cells, the BZLF1 was not switched off (data not shown), suggesting that the viral genome had already been epigenetically altered in such a way that could not be undone by Notch ligation.

As BZLF1 expression in BNHL-1 cells followed the downregulation Zeb2 expression after the removal of Notch ligation, we asked if Zeb2 is involved in BZLF1 inhibition in primary B cell infection. Contrary to our expectations, we found that the transcription of both Zeb1 and Zeb2 mRNA was rapidly downregulated in the newly infected B cells, irrespective of whether or not they were cocultured on the OP9-DLL1 cells (Fig. 5B and C). One possible explanation for this anomaly is that the amount of BZLF1 transcription observed in primary infection was so low that it could have been due to less than 0.2% of cells expressing BZLF1. In such a scenario, measurement of Zeb1 and Zeb2 expression in the total population is uninformative.

The major difference between infection of BNHL-1 cells and infection of normal primary resting B cells is that primary B cells go on to express the full growth transformation program of EBV genes (i.e., latency III). We reasoned that an EBV gene expressed early following infection may be responsible for the rapid downregulation of Zeb2 following infection. Since both EBNA2 and Notch function through RBP-Jκ, we examined Zeb1 and Zeb2 expression in primary resting B cells infected with virus with an EBNA2 deletion. As shown in Fig. 5D, we observed that Zeb2 mRNA transcription was maintained in B cells infected with virus with an EBNA2 deletion. Exactly the same observation was made for Zeb1 mRNA transcripts (not shown). This suggests that EBNA2 may play a role in inhibiting Zeb2 expression, even in the presence of Notch ligation.

### Notch ligation inhibits LMP1 expression.

**FIG 6:**
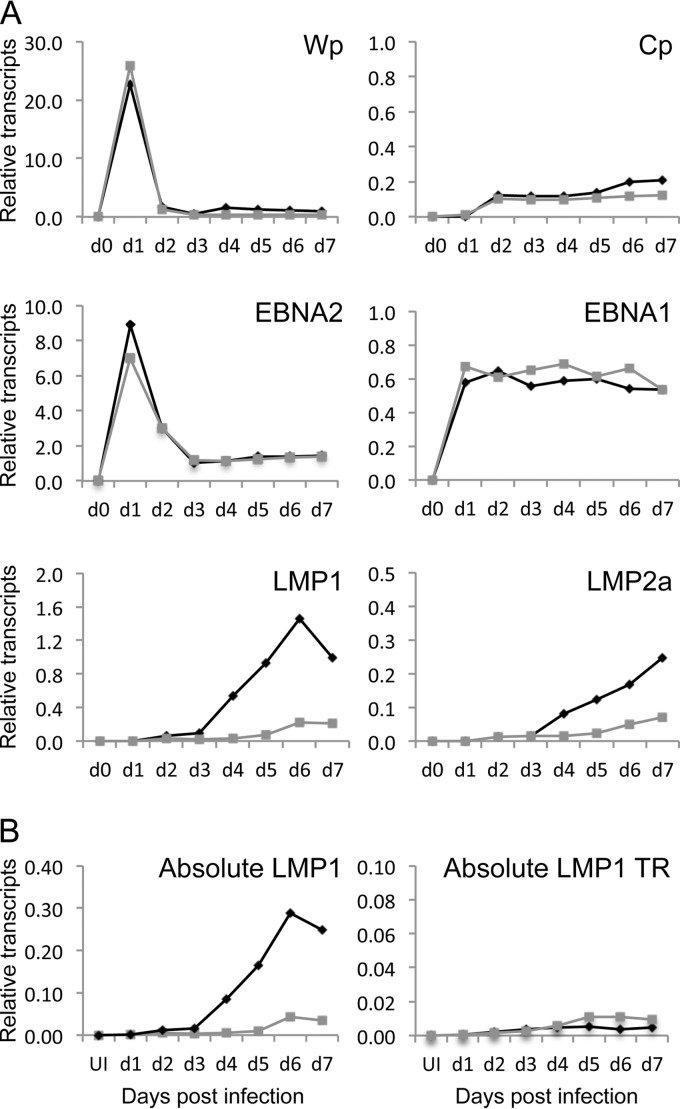
Notch ligation inhibits LMP1 expression in primary B cell infection. (A) Quantitation of relative levels of EBV latent gene transcripts. Primary B cells were infected and cocultured on OP9-DLL1 stromal cells or on OP9-GFP stromal cells. The graphs show an example of the normalized amounts of the transcripts for each of the latent genes expressed early following primary B cell infection. All infections were performed four times. (B) Quantitation of the absolute amounts of LMP1 mRNA transcripts. qRT-PCR was performed for total LMP1 or TR (LT-R1 promoter)-derived LMP1 mRNA transcripts, and the amounts were compared with those of a serially diluted plasmid of known copy number encoding the LMP1 amplicon sequences. Both graphs show representative values for each LMP1 transcript either on OP9-DLL1 cells or on OP9-GFP cells. All assays were performed in triplicate with three biological replicates. Representative plots are shown. UI, uninfected.

EBNA2 is one of the first latent proteins to be expressed during infection of normal B cells and is an important transcriptional regulator of other latent EBV genes. Given the previously reported interrelationships between EBNA2 and Notch signaling pathways, we therefore queried whether Notch ligation might modulate the pattern of latent gene expression during primary infection. We performed qRT-PCR for the EBV genes expressed during infection of resting B cells cocultured on OP9-DLL1 or OP9-GFP stromal cells or in suspension. Figure 6A shows representative examples of the transcription of mRNA for the latency III-associated growth-transforming EBV genes. Transcription from the Wp and Cp promoters, including the transcription of EBNA1 and EBNA2, was unaffected by Notch ligation. However, the levels of LMP1 mRNA were consistently more than 5-fold lower when the B cells were cocultured on OP9-DLL1 cells. Similarly, the levels of LMP2a mRNA were at least 2- to 3-fold lower on OP9-DLL1 cells.

LMP1 is regulated by two promoters: the classical ED-L1 promoter containing an EBNA2-regulated element and a second promoter, LT-R1, within the terminal repeat (TR) region of the EBV genome that lacks this regulatory element. During primary B cell infection *in vitro*, the predominant promoter used for LMP1 expression is the classical EBNA2-regulated promoter. We therefore examined which LMP1 promoter was being used and which was being inhibited by Notch ligation during primary B cell infection. To this end, we quantitated the relative number of LMP1 mRNA transcripts using two different assays and a uniform standard; one assay measured the total number of LMP1 transcripts from either promoter, and a second measured only the number of LMP1 transcripts from the LT-R1 promoter within the TR. Figure 6B shows the results of one representative assay of three replicate experiments. By day 6 postinfection, the total number of LMP1 mRNA transcripts was reduced from 0.28 transcript per B2M transcript when cells were cocultured on OP9-GFP cells to 0.042 transcript per B2M transcript when cells were cocultured on OP9-DLL1 cells. Interestingly, the number of transcripts of LMP1 derived from the promoter in the TR increased to an average of 0.01 per B2M transcript by day 6 postinfection. These results suggest that Notch ligation strongly inhibits the transcription of LMP1 mRNA originating from the ED-L1 promoter containing the EBNA2-regulated element.

**FIG 7:**
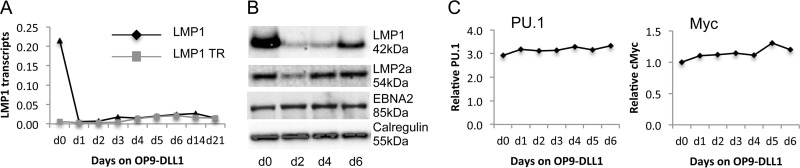
Notch ligation inhibits LMP1 expression in LCLs. (A) Quantitation of absolute amounts of total LMP1 and TR (LT-R1 promoter)-derived LMP1 mRNA transcripts in LCLs cocultured on OP9-DLL1 stromal cells. qRT-PCR was performed for total LMP1 and TR (LT-R1 promoter)-derived LMP1 mRNA transcripts, and the amounts were compared with those of a serially diluted plasmid of known copy number encoding the LMP1 amplicons. The graph shows the results of a representative assay of the time course of LCLs on stroma. The assay was performed in triplicate on four biological LCL replicates. (B) Western blot analysis of LMP1 expression in LCLs cocultured on OP9-DLL1 stromal cells. The blots were probed with LMP1, LMP2a, and EBNA2 antibodies. The blots show the inhibition of LMP1 expression, a temporal drop in LMP2a expression which rebounded by 4 days, and no change to EBNA2 expression. (C) Relative qPCR of PU.1 and MYC in LCLs cocultured with OP9-DLL1 stromal cells. There was no change in mRNA transcripts following culture on OP9-DLL1 stromal cells.

Given that Notch ligation inhibits ED-L1-driven LMP1 transcription during primary cell infection, we asked if Notch ligation could switch off LMP1 transcription once the B cells have transformed into LCLs. We therefore cocultured established LCLs with OP9-DLL1 cells or with the control OP9-GFP cells and examined LMP1 transcription daily. Figure 7A shows the relative quantitation of total LMP1 mRNA transcripts and TR-derived LMP1 mRNA transcripts over 21 days of coculture on OP9-DLL1 cells. Remarkably, a 30-fold reduction in total LMP1 mRNA levels was observed within 24 h of DLL1 exposure (Fig. 7A), but no such decrease was observed when the LCL was cocultured with OP9-GFP cells (not shown). Interestingly, there was a corresponding increase in the amount of TR-derived LMP1 transcripts, to the extent that most of the LMP1 transcripts in the LCL cells grown on OP9-DLL1 were probably derived from the TR promoter instead of the classical EBNA2-regulated LMP1 promoter. The drop in total LMP1 transcripts was mirrored by a drop in LMP1 protein levels (Fig. 7B), which was maintained for at least 21 days (data not shown). However, LMP1 protein levels rapidly rebounded to the levels observed without Notch ligation if the B cells lost contact with the OP9-DLL1 cells, which was observed on day 6. Examination of the LMP2A and EBNA2 proteins (Fig. 7B) and transcripts (not shown) following coculture with OP9-DLL1 cells showed an initial drop in LMP2a expression at day 2 which quickly recovered to the levels observed when the cells were not cocultured with OP9-DLL1 stroma.

Finally, we addressed whether the drop in LMP1 expression was caused by the specific inhibition of EBNA2 induction of LMP1 by Notch or if all EBNA2-regulated genes were affected. To this end, we first examined whether other transcription factors that regulate LMP1 expression from the classical promoter were downregulated by Notch ligation. We performed a qRT-PCR assay for PU.1, a transcription factor essential for LMP1 expression, which revealed that the level of mRNA transcripts remained unaffected by Notch ligation compared to the effect of no ligation (Fig. 7C). We also performed a qRT-PCR assay for Myc, which is a specific downstream target of EBNA2, to confirm that Notch ligation did not inhibit all downstream targets of EBNA2 (Fig. 7C).

## DISCUSSION

Little is known about the regulation of EBV gene expression following primary infection of resting B lymphocytes *in vivo*, particularly how EBV gene expression is downregulated from the latency III gene expression profile to the latency 0 profile seen in circulating resting memory B cells. Much of our understanding about the regulation of individual viral genes has come from studies of cell lines. Although they are extremely informative, such studies have limitations in that they do not necessarily take into account signaling from the microenvironment *in vivo*, be it mediated by contact with neighboring cells of different lineages or by soluble factors released from these cells. With regard to soluble factors, it has been shown by others that the T cell-derived cytokines interleukin-21 (IL-21) and CD40L may downregulate EBNA2 expression in latency III LCLs and that IL-4, IL-10, IL-13, and IL-21 can induce LMP1 expression in certain malignant B cell lines (41–44). In our study, we investigated the consequence of Notch ligation in EBV-infected B cells and observed that while DDL1/Notch-2 activation does not alter the expression of EBNA2, it markedly inhibits the ability of EBNA2 to induce LMP1 and LMP2a transcription during primary B cell infection and in established LCLs. Furthermore, activation of Notch-2 also inhibits lytic cycle induction through the upregulation of Zeb2 expression, which represses the transcription of BZLF1.

The control of EBV latency and lytic reactivation in EBV is a complex process involving a number of transcriptional activators and repressors acting upon the immediate early transducers of lytic cycle activation, BZLF1 (Zta) and BRLF1 (RTA). The master regulator of the latent-lytic cycle switch, BZLF1, binds directly to Zta response elements throughout the viral genome and activates both early and late lytic cycle genes (45). BZLF1 does not rely on DNA binding proteins, such as RBP-Jκ, to activate the promoters of the lytic cycle genes, so it is not regulated by Notch at this level. Four transcription factors have previously been shown to regulate BZLF1 and BRLF1 either positively or negatively; these include Zeb1 and Zeb2 (40, 46–48) and Oct-1 and Oct-2 (38, 39). The Zeb1 and Zeb2 transcription factors inhibit lytic cycle reactivation by repression of BZLF1 transcription; both Zeb1 and Zeb2 bind to the ZV element-containing region of the Z promoter (Zp; the BZLF1 promoter) and repress transcription initiated at Zp (40). Oct-2 inhibits lytic cycle reactivation by directly interacting with BZLF1, resulting in the attenuation of BZLF1 binding to lytic viral promoters. Conversely, Oct-1 promotes viral lytic reactivation by interacting with BRLF1 to enhance the interaction of BRLF1 with viral lytic promoters. These transcription factors are not all functional in the same cell lineage; indeed, their activities appear to be cell type specific (38–40, 46–48). Our present work shows that DLL1/Notch-2-mediated inhibition of BZLF1 expression in the EBV-infected BNHL-1 cell line is mediated via the Notch-regulated expression of the transcription factor Zeb2.

A role for Notch in the regulation of the virus lytic cycle has not previously been described for EBV. In contrast, the lytic cycle of Kaposi's sarcoma-associated herpesvirus (KSHV) is known to be regulated both positively and negatively by the Notch pathway, although mechanistically the roles of Notch in regulating the EBV and KSHV lytic cycle are quite distinct. The master regulator of KSHV lytic cycle is the virally encoded RTA (ORF50), a transcription factor which can stimulate the transcription of up to 80 lytic genes (49). Although RTA can bind to a specific DNA sequence found in a few viral promoters, for the most part RTA relies on cellular coregulators, including RBP-Jκ (CSL) (50), C/EBPα (51, 52), Oct-1 (53), and c-Jun (54), to bind to DNA. For instance, RTA forms a complex with RBP-Jκ and binds to RTA-responsive promoters with RBP-Jκ binding sites, recruits coactivators through its own C-terminal activation domain, and activates transcription of that gene. In some cases this is directly influenced by cell surface Notch ligation, as is the case for K2 and K5 (55).

Our observations on the control of EBV lytic cycle entry in the infected BNHL-1 lymphoma cell model provide novel insight into the regulation of lytic cycle induction by extracellular signals. We also observed that Notch-2 ligation inhibited the very low levels of BZLF1 transcription that occur during *in vitro* infection of primary B cells. However, the effect of this small but reproducible effect warrants closer examination to determine its significance. Unexpectedly, in contrast to the findings presented in one previously published report (37), we found that EBV infection itself caused a substantial downregulation of Zeb2 expression which was probably EBNA2 dependent and was apparently not reversed by Notch ligation. This indicates that the downregulation of Zeb2, even if necessary, is not by itself sufficient to induce BZLF1 expression. It is not clear by what mechanism Notch-2 activation inhibits what little BZLF1 transcription does occur. As the amount of BZLF1 mRNA detected is equivalent to that from less than 0.5% of cells entering the lytic cycle, it is possible that Notch-2 activation may be upregulating Zeb2 expression in a relevant subset of cells able to transcribe BZLF1; alternatively, Notch-2 activation may be acting via a different mechanism in primary infection of normal B cells.

A more pronounced consequence of Notch activation during infection of normal B cells and in established LCLs was that it inhibited the *de novo* expression of LMP1 in the primary infection and switched off LMP1 expression in established LCLs already expressing LMP1. One explanation for this observation suggests that the interaction of ICN-2 with RBP-Jκ blocks the interaction of EBNA2 with RBP-Jκ, thereby inhibiting the initiation of LMP1 transcription from the classical LMP1 promoter, which is EBNA2 responsive (56). In support of this hypothesis, quantitative PCR assays revealed that the residual LMP1 expression in the presence of Notch activation was actually initiated from the LMP1 promoter within the terminal repeats, which is not EBNA2 responsive.

Similarly, Notch ligation also inhibits *de novo* LMP2a transcription during primary B cell infection and downregulates LMP2a expression in an established LCL. Like LMP1, the promoter for LMP2a is also EBNA2 responsive (16), suggesting that expression should remain low if LMP2a behaves like LMP1 following Notch ligation. However, unlike LMP1 transcription and expression in Notch-activated LCLs, the inhibition of LMP2a was transient and transcription and expression subsequently recovered to the levels observed without Notch ligation. This observation suggests either that LMP2a is not regulated by Notch or that EBNA2 overrides this signal. However, LMP2a is often detected in the absence of EBNA2 in B cell lymphomas, such as Hodgkin's lymphoma. LMP2a has been demonstrated to induce its own promoter, and several reports have demonstrated that LMP2a constitutively activates the Notch pathway (57–59). Furthermore, ICN can bind to and activate the LMP2a promoter (60), and deletion of the RBP-Jκ consensus sequences results in a significant decrease in LMP2a promoter activity (57). LMP2a therefore appears to utilize the Notch pathway to induce its own expression in the absence of EBNA2, which offers an explanation for why Notch activation has subtly different effects on LMP1 and LMP2a expression.

If LMP2a is indeed constitutively activating the Notch pathway to induce its own expression in Hodgkin's lymphoma, our results would suggest that either LMP1 expression in these cells would be inhibited or LMP1 expression would be initiated at the terminal repeat promoter. However, the promoter origin of LMP1 expression in Hodgkin's lymphoma does not appear to have been elucidated. One study identified TR-derived LMP1 transcripts in 10 out of 12 cases of Hodgkin's lymphoma, albeit with concurrent expression from the classical promoter (61). However, these assays were performed on total RNA extracted from Hodgkin's lymphoma biopsy specimens using endpoint PCR, thereby clouding the issue.

This study has extended the possible mechanisms by which EBV-infected B cells in normal lymphoid tissues might be prevented from activating lytic virus replication and switching off growth transformation-associated latent viral genes during the process of establishing latency 0 in nonproliferating memory B cells. Previous studies (43, 44) identified a potential and significant role for T cell-derived cytokines and soluble CD40 ligand in downregulating the expression of EBNA2, albeit with a concomitant upregulation of LMP1. To that body of work, we now add the potential of stromal cell interactions to dramatically modulate viral gene expression. The effects of DDL1-mediated Notch-2 activation in infected B cells are remarkable and include interference with EBNA2 function, the transcriptional downregulation of LMP1 and LMP2, and the inhibition of lytic virus replication through the prevention of BZLF1 induction. We consider it likely that there will be additional, yet to be identified stromal interactions which modulate viral gene function or expression. The next challenge will be to delineate which of the many possible extracellular signaling events are actually crucial for the normal process of establishing the latency 0 state in memory B cells and how the various cellular interactions at different microanatomical sites in the lymphoepithelial tissue might enable reactivation of lytic virus replication for secretion into the oropharynx.
